# An SNP-based second-generation genetic map of *Daphnia magna* and its application to QTL analysis of phenotypic traits

**DOI:** 10.1186/1471-2164-15-1033

**Published:** 2014-11-27

**Authors:** Jarkko Routtu, Matthew D Hall, Brian Albere, Christian Beisel, R Daniel Bergeron, Anurag Chaturvedi, Jeong-Hyeon Choi, John Colbourne, Luc De Meester, Melissa T Stephens, Claus-Peter Stelzer, Eleanne Solorzano, W Kelley Thomas, Michael E Pfrender, Dieter Ebert

**Affiliations:** Zoologisches Institut, Universität Basel, Vesalgasse 1, 4051 Basel, Switzerland; Department of Computer Science, University of New Hampshire, Durham, NH 03824 USA; Department of Biosystems Science and Engineering, ETH Zurich, 4058 Basel, Switzerland; Laboratory of Aquatic Ecology, Evolution and Conservation, University of Leuven, Charles Deberiotstraat 32, B-3000 Leuven, Belgium; Department of Biostatistics, Georgia Regents University, Augusta, GA 30912-4900 USA; School of Biosciences, The University of Birmingham, Birmingham, B15 2TT UK; Department of Biological Sciences, Galvin Life Science Center, Notre Dame, IN 46556 USA; Universität Innsbruck, Forschungsinstitut für Limnologie, 5310 Mondsee, Austria; Hubbard Center for Genome Studies, University of New Hampshire, Durham, NH 03824 USA; Peter T. Paul College of Business and Economics, University of New Hampshire, Durham, NH 03824 USA; Department of Zoology, George S. Wise Faculty of Life Sciences, Tel Aviv University, Tel Aviv, Israel; School of Biological Sciences, Monash University, Melbourne, Victoria 3800 Australia

## Abstract

**Background:**

Although *Daphnia* is increasingly recognized as a model for ecological genomics and biomedical research, there is, as of yet, no high-resolution genetic map for the genus. Such a map would provide an important tool for mapping phenotypes and assembling the genome. Here we estimate the genome size of *Daphnia magna* and describe the construction of an SNP array based linkage map. We then test the suitability of the map for life history and behavioural trait mapping. The two parent genotypes used to produce the map derived from *D. magna* populations with and without fish predation, respectively and are therefore expected to show divergent behaviour and life-histories.

**Results:**

Using flow cytometry we estimated the genome size of *D. magna* to be about 238 mb. We developed an SNP array tailored to type SNPs in a *D. magna* F2 panel and used it to construct a *D. magna* linkage map, which included 1,324 informative markers. The map produced ten linkage groups ranging from 108.9 to 203.6 cM, with an average distance between markers of 1.13 cM and a total map length of 1,483.6 cM (Kosambi corrected). The physical length per cM is estimated to be 160 kb. Mapping infertility genes, life history traits and behavioural traits on this map revealed several significant QTL peaks and showed a complex pattern of underlying genetics, with different traits showing strongly different genetic architectures.

**Conclusions:**

The new linkage map of *D. magna* constructed here allowed us to characterize genetic differences among parent genotypes from populations with ecological differences. The QTL effect plots are partially consistent with our expectation of local adaptation under contrasting predation regimes. Furthermore, the new genetic map will be an important tool for the *Daphnia* research community and will contribute to the physical map of the *D. magna* genome project and the further mapping of phenotypic traits. The clones used to produce the linkage map are maintained in a stock collection and can be used for mapping QTLs of traits that show variance among the F2 clones.

**Electronic supplementary material:**

The online version of this article (doi:10.1186/1471-2164-15-1033) contains supplementary material, which is available to authorized users.

## Background

One hundred years ago, A.H. Sturtevant [1] introduced a new concept in genetics, namely the mapping of genes by three-factor test crosses. Genes were marked by discrete phenotypic traits, and their order on a chromosome was deduced by their recombination rates. This type of linkage mapping remains the dominant method of determining the arrangement of genes on chromosomes today. Phenotypic markers were later replaced by molecular genetic markers to achieve today’s high resolution mapping of large numbers of single nucleotide polymorphisms (SNPs) [2, 3]. Advances in molecular genetics and genomics offer an unmatched opportunity for producing genetic maps and extending them to include phenotypic traits of species without prior genomic knowledge. Indeed, it has become relatively easy to produce medium- to high-density maps, even for organisms not typically considered to be genetic model systems [4–6]. This ability is particularly valuable for species, in which ecologically relevant phenotypes should be linked to the underlying genetics e.g. [4, 7, 8]. This possibility of linking ecologically relevant phenotypes with their underlying genes and genetic architecture has spurred a tremendous amount of research in fields as different as epidemiology, ecology, developmental biology, medicine, agriculture and aquaculture [9–12].

A biological system celebrated for the insights it has enabled in ecology and evolution are the waterfleas of the genus *Daphnia* (Crustacea: Cladocera) [13–17]. *Daphnia* are planktonic freshwater crustaceans common in ponds and lakes around the world [18]. Their cyclic parthenogenetic mode of reproduction makes it possible to produce genetic crosses among lines and keep clonal lines for many generations, so that the same genotype can be replicated in the lab, which reduces the error variance when testing traits under the same environmental conditions [19]. *Daphnia* have played an important role in elucidating important biological phenomena such as phenotypic plasticity (including predator-induced defence), life history evolution, sexual reproduction, immune defence against parasites, and ecotoxicology [13–16, 20, 21].

Two *Daphnia* species of particular interest in the field of environmental genomics, *D. magna* and *D. pulex*, have had a number of genetic and genomic tools developed for them, including genome sequences, EST libraries, RNAi, and transgenesis [22–25]. Low-resolution linkage maps are also available for these two species [26, 27], offering some possibility of mapping phenotypic traits. However, these maps are not sufficiently dense to help assemble the *Daphnia* genomes, which still consist of several thousand scaffolds and contigs. Thus, while we know more about the ecology of *Daphnia* than any other taxon, comparatively little is known about their genetics. This situation is changing, however, with the recent burst of methods using next generation sequencing of model organisms, which has enabled the development of high-density linkage maps [2, 28]. One method for producing these post-genomics linkage maps is with SNP micro arrays, which use fixed sets of predetermined SNPs in a crossing scheme. The objective of this study was to apply this technique to *D. magna*, building an SNP array-based genetic map of the species and to analyse the genetic basis of quantitative genetic traits. We used an existing, clonally maintained F2 QTL panel of *D. magna*[27, 29] and a SNP microarray based on SNPs derived from the F1 hybrid clone of the panel.

*Daphnia* populations are known to exhibit marked population divergence, often in line with local adaptation [30–36]. Predation by fish is a strong agent in selecting for local adaptation and has been shown to lead to shifts of *Daphnia* communities and populations towards smaller *Daphnia* species, reduced body size, earlier maturation and to influence behavioural anti-predator response within species such as increased diel vertical migration [14, 32, 34, 37, 38]. The parent clones used in our QTL F2 panel were collected from habitats with strongly divergent ecology: a fishless rock pool in Finland and a carp breeding pond in Germany, respectively. By mapping the life history and behavioural traits of 193 F2 genotypes from these parent clones on the genetic map produced here, we are able to gain insight in the genetic architecture of these traits. We expect to find that alleles from the German parent clone (habitat with fish) code for smaller body size and lower reproductive effort, and shift position towards deeper water in the presence of fish kairomones.

## Methods

### *Daphnia*clones and the F2 panel

We constructed an F2 panel using two parental strains, one from a pond near Munich, Germany (geographic coordinates: 48.206375, 11.709727), and the other from a rock pool on a skerry island near Tvärminne, Finland (59.833183, 23.260387) [27]). The Munich clone, which does not easily reproduce sexually, was selfed one time, resulting in the Iinb1 clone, whereas the Finnish clone, which reproduces easily sexually, was selfed three times, resulting in the Xinb3 clone. A Xinb3 female and an Iinb1 male were crossed to produce the F1 hybrid clone. Because sex is environmentally determined in *Daphnia* and they have no sex chromosomes, the same genotype can be either male or female, allowing selfing of genotypes. A single F1 clone was maintained as a clonal line and selfed to produce F2 offspring, here called the F2 panel. The F2 panel consisted of 353 clones, 198 of which belonged to the core panel used for the first generation genetic map [27] and additional 155 clones of which were in an extended panel. All F2 clones are maintained as clonal lines and can be used to detect the genetic basis of any trait variation between the two parental clones, or between F2 clones.

The *D. pulex arenata* clone, which was used in the *D. pulex* genome project [22], was originally obtained from the Indiana University, USA stock centre, but has been kept in the Basel University Daphnia stock centre since 2006 by means of asexual reproduction.

Handling Daphnia species and their parasites does not require specific permissions.

### Genome size estimation with flow cytometry

We used a detergent-trypsin method for flow cytometric analysis of genome size [39]. Prior to the preparation of tissues *Daphnia* females were starved in ADaM medium [40] containing Sephadex beads. Starved females were then washed in few millilitres of stock solution (3.4 mM Trisodium citrate dihydrate, Nonidet P40 at 0.1% v/v, 1.5 mM Sperminetetrahydrochloride, 0.5 mM Trishydroxymethyl-aminomethane, pH 7.6). For each replicate, we homogenized ten females in 350 μl stock solution using a 1 ml-dounce tissue homogenizer with 20 strokes ("tight" pestle). Large debris was removed by filtration through a 35 μm mesh nylon sieve. An aliquot of 100 μl of the homogenized tissue suspension was digested by addition of 450 μl of 0.003% Trypsin (dissolved in stock solution) for 10 min at room temperature. To prevent further degradation, 0.05% trypsin inhibitor was added (this solution also included 0.01% RNAse A) and the samples were incubated for another 10 min. Finally, samples were stained with propidium iodide at a concentration of 50 μg/ml. Stained samples were kept for at least 3 h (or, overnight) on ice in the dark. Flow cytometric analysis was performed with a FacsCalibur flow cytometer (BD Biosciences) at an excitation wavelength of 488 nm. Propidium iodide emission was measured in the FL2-A channels according to the manufacturer’s instructions. As internal standard of known genome size we used the fruit fly, *Drosophila melanogaster* (strain ISO-1, nuclear DNA content: 0.35 pg; [41]). Briefly, ten female *Drosophila* heads were homogenized in 0.5 ml stock solution with 15 strokes in the Dounce homogenizer, and 100 μl of this homogenate was co-prepared with the *Daphnia* samples and stained in exactly the same way. Since the diploid peak of *D. pulex arenata* (the clone used in the *D. pulex* genome project [22]) strongly overlapped with the *Dro. melanogaster*, it was necessary to use the *Daphnia magna* clones (Iinb1, Xinb3) as a further (internal) standards for *D. pulex arenata*. All samples (focal and standards) were first analysed separately on the cytometer, to identify the position of the 2C peaks and to determine the approximate concentration of nuclei, and were then measured in combinations. Single samples were run until a pre-specified number of 7,000 events (i.e., particles registered by the fluorescence detectors) were reached, typically at a rate ~30 events per second. For combined samples (=focal species + internal standard) the number of events was increased to 15,000. At least five biological replicates were prepared for each *Daphnia* clone. To avoid a bias due to temporal fluctuations in flow cytometer performance, each replicate of the same isolate was measured on a different day (sometimes with several weeks between two measurements). Coefficients of variance (CVs) of individual peaks typically ranged between 2.5% and 5% for both *Drosophila* and *Daphnia*. Very few measurements had CVs higher than 6%, and those replicates were discarded. Conversion from picograms DNA to base pairs were made with the factor: 1 pg =978 mb [42].

### DNA isolation

DNA was isolated using the modified CTAB (cetyltrimethylammonium bromide) method: CTAB homogenization buffer (HB) 200 ml; water 160 ml, NaCl 16.36 g, 14.3 M 2-mercaptoethanol 400 μl, 1 M Tris–HCl (pH = 8) 20 ml, 0.5 M Na_2_EDTA (pH = 8) 8 ml and CTAB 4 g. About 20 adult *D. magna* animals were placed into a clean 1.5 ml tube. 200 μl of warm (~65°C) HB is added immediately. Animals were ground and additional 300 μl of HB was added. We incubated at 65°C for 1 hour and added 1 μl of (100 mg/ml) RNase A. Then we incubated at 37°C for 40 minutes. The supernatant was pipetted into a phase-lock gel tube (Eppendorf 955154045). We proceeded with standard phenol/chloroform extraction.

### SNP discovery and probe design

The *D. magna* single nucleotide polymorphism (SNP) array was specifically designed to incorporate the 3,370 scaffolds and 37,774 contigs from the incomplete draft *D. magna* assembly v2.4, 20100327 ( http://wfleabase.org/). The first step in developing the SNP array was to identify SNPs in the F1 clone. F1 clone DNA was sequenced using single-read Illumina sequencing to a coverage depth of 37.64 (s.d. ±15.45). Reads were mapped to the *D. magna* reference assembly and SNPs detected by using novoalign (available at http://www.novocraft.com). We filtered the SNPs using four criteria: 1) SNPs that fell into regions outside of one standard deviation from the coverage mean were removed. Sites that are underrepresented can provide false positive SNP calls, while sites that are overrepresented have the potential to be paralogous. 2) SNPs that contained average quality or mapping scores less than 20 were removed, ensuring SNP base call accuracy of 99%. 3) Using megablast [43], the reference contigs were mapped to the reference scaffolds. Contigs that contained greater than 80% identity to any scaffold were removed from the analysis. This ensured that the contigs used in the SNP array were not misassembled alleles or paralogous from the original reference assembly of the *D. magna* genome. This procedure resulted in 417,862 scaffold SNPs, and 44,110 contig SNPs on 96.1% of the scaffolds and 28.5% of the contigs.

The 461,972 SNPs were passed to Roche NimbleGen, Inc. (Madison, WI) which generated forward and reverse probes with each nucleotide combination (A, C, T, G) for a total of 3,695,776 probes. NimbleGen used proprietary software to filter the SNPs for uniqueness in array development, reducing the number of SNPs to 3,196,942 scaffold probes and 240,406 contig probes covering 95.7% of the scaffolds and 26.1% of the contigs.

From the complete probe sets (eight probes at a locus), we used the EMBOSS suite [44] to select for ideal probe conditions of G/C content less than 50% and melting temperature between 55°C and 72°C. For the remaining 2,626,168 scaffold probes and 197,320 contig probes, we randomly selected one probe set (eight probes) for each contig and two probe sets (16 probes) for each scaffold when available. If multiple probes were available for a specific contig or scaffold, we selected the probe that had the highest minor allele frequency (closest to 50/50 allele frequency). In order to cover the largest scaffolds more thoroughly, we selected from the largest 1,000 scaffolds an additional marker that was furthest in physical distance from the previously selected marker. This selection process generated 64,168 (8,021 loci) scaffold probes and 69,216 (8,652 loci) contig probes for a total of 133,384 (16,673 loci) probes. The final probe set contained at least one marker on 94.1% (3,171/3,370) of the scaffolds and 22.9% (8,652/37,774) of the contigs. An additional 269 probes of biological interest [45] were added to the final probe set (133,653 total probes).

### SNP array hybridizations

Labelled genomic DNA was synthesized using a NimbleGen Dual-Color DNA Labeling Kit (Roche NimbleGen, Inc., Madison, WI). The labelling reaction contained 500 ng of gDNA and 0.5 OD of Cy-labelled random nonamers. This mixture was heat denatured at 98°C for 10 minutes and promptly chilled in an ice water bath. 10 mM dNTP mix and 50U of Klenow Fragments were added to each sample to attain a total reaction volume of 50 μl. The reaction was incubated for 2 h at 37°C, and terminated by the addition of 0.5 M EDTA. The product was precipitated in isopropanol, rinsed in cold 80% ethanol, and desiccated using a Savant DNA 120 SpeedVac Concentrator (ThermoScientific, Waltham, MA). Pellets were resuspended in nuclease free water. Cy-labeled product was quantified using a Nanodrop ND-2000 (ThermoScientific), and 30 μg (15 μg each of Cy-3 and Cy-5) labelled product was removed, pooled, and desiccated for subsequent hybridization to the array.

The labelled product was resuspended using Sample Tracking Controls (Roche NimbleGen, Inc.). Components from the Hybridization Kit, LS (Roche NimbleGen, Inc.) were combined to prepare the hybridization solution. This solution was added to each subarray pool for a total volume of 8ul. The product was then denatured at 95°C for five minutes. Hybridization was performed on a NimbleGen Hybridization System (Roche NimbleGen, Inc.) following manufacturer’s guidelines at 42°C for 20–22 hr. The post hybridization wash was conducted using a Wash Buffer Kit (Roche NimbleGen, Inc.). Arrays were agitated consecutively in Wash Buffer I (2 minutes), Wash Buffer II (1 minute), and Wash Buffer III (15 seconds). The arrays were dried for one minute using a High-Speed Microarray Centrifuge (Arrayit Corp., Sunnyvale, Ca.). Image acquisition was attained using the NimbleGen MS 200 Microarray Scanner (Roche NimbleGen, Inc.) at 2 μm resolution. Photomultiplier tube (PMT) Gain was adjusted automatically to ensure uniform intensities and acquire the best image for each subarray.

### SNP filtering

A method of analysing custom NimbleGen arrays was developed to predict genotypes for each putative SNP on the array. In the first step, spatial smoothing was conducted on the intensity values for each array to reduce within array technical bias following the approach of Wang et al. [46] using 256 zones. To evaluate the genotype of each locus, we then calculated a parameter (*homoIndex).* This index was based on spatially smoothed fluorescent intensities (four alternative SNP versions for each of the two strands). Each homoIndex value determined the distribution of four strand-specific oligos, creating a value for each strand independently. The smoothed intensities (r1 to r4) for each strand oligo are sorted in decreasing order and used in the homoIndex equation.


Where r1 is the highest signal intensity and r4 is the lowest. The ideal values of *h* will be 1 for heterozygous loci where two oligos represent the two alleles (r1 and r2) have equal signal strength, and zero where there is only a single allele (r1). If the classification for a group of four readings is homozygous, the r1 oligo represents the allele (nucleotide) on both chromosomes in the associated strand. If the classification for a group of four readings is heterozygous, the r1 and r2 oligo represent the two alleles present. Default parameters for the homoIndex are less than 0.475 for homozygous loci and greater than 0.525 for heterozygous loci.

Following this step, several filters were applied to identify the loci with the highest and most consistent genotyping. First, the complementary genotypes predicted by the homoIndex values both had to predict the same genotype and complementary nucleotide(s). Second, these nucleotides predicted in any F2 had to match one or both parental genotypes. Any locus that did not meet these criteria was marked as invalid data. Third, we required that the array correctly genotypes a given SNP as heterozygote in the F1 hybrid clone on eight replicate arrays. Similarly, we ran replicate arrays on the parental clones and rejected loci that were not correctly genotyped.

After those filters were applied, the homoIndices for each SNP across all F2 clones (F1 and parents excluded) were subjected to a DIP test for bimodal data distribution [47] using the R "diptest" package [48]. The *Dip Test* step executes two dip tests per SNP, one for each strand-specific homoIndex. Each dip test generates a *p-value* indicating the strength of the bimodal distribution. P-values less than 0.2 were passed, and genotypes were passed to the next pipeline step. Finally, datasets of F2 clones with more than 25% of their genotypes invalid were rejected. In most cases, fresh DNA from these F2 clones was processed again on the array.

### Map building

A total of 353 clonal lines were used to construct the linkage map. After purging the loci that did not meet our quality criteria, 1,324 markers remained. All the statistics were done in the R environment (version 2.9.2), and linkage groups were constructed with the R package R/qtl (version 1.13-7) [49]. Initial marker order was determined with the command "formLinkageGroup" and refined with the command "ripple" with a seven markers window.

### Life history experiment

We used individual females from a subset of 193 F2 clones to conduct an experiment assessing variation in body size and lifetime fecundity. To minimise maternal effects, we began with three independent replicates of each F2 clone, using 1- to 3-day-old females that were raised individually in 100-ml jars for two generations. From the third generation, we chose one individual from the third-clutch of each replicate line and followed these individuals until death. Each individual was placed in a 100-ml jar filled with *Daphnia* medium (ADaM). Every three days thereafter, we moved the individuals to new jars containing 80-ml of fresh medium and counted the number of offspring produced. We recorded the age at which the first clutch was deposited into the brood pouch and, on day 28, measured body size (the distance from the upper edge of the head to the base of the tail spine) under a dissecting microscope.

All generations of animals were maintained in a climate-controlled incubator (light/dark: 16/8 h; 20 ± 0.5°C), with their shelf location regularly shuffled to equalise any positional effects. To meet the increased food requirements of growing *Daphnia*, we upped daily food ratios from 0.5 × 10^6^ algae cells of *Scenedesmus obliquus* per *Daphnia* on day 2, to 1 × 10^6^, 2 × 10^6^, 2.5 × 10^6^, 3 × 10^6^ and 5 × 10^6^ algal cells per animal per day on days 4, 7, 9, 11 and 13, respectively.

### Quantification of phototactic behaviour

Phototactic behaviour was quantified in the presence and absence of fish kairomones using the experimental set-up described by De Meester [19, 50]. Briefly, the *Daphnia* clones were cultured under standardized conditions (1-l bottles, 20 ± 2°C, 14-h light/10-h dark photoperiod, 20 adults liter^-1^; high food concentration: 1.5 10^5^*Scenedesmus obliquus* cells ml^-1^, adjusted daily), in either dechlorinated tap water (aged 24 h) or in dechlorinated tap water conditioned by fish kairomones. The latter medium was prepared by allowing two ide (*Leuciscus idus*; Teleostei, Cyprinidae; approx. 8 cm standard length) to swim in the medium for 24 hours, after which the water was filtered over 0.4 μm and diluted 10×. The fish were fed *Daphnia* in a separate aquarium so that the medium remained free of alarm pheromones. To quantify phototactic behaviour, we used an experimental glass column (25 cm high, 5 cm internal diameter). The bottom of the column was covered with small black aquarium pebbles to reduce light reflection, and the column was filled with dechlorinated tap water and positioned in a dark box in a temperature-controlled room (20 ± 2°C). The column was fitted with a 150 W fibre light source, with the end of the fibre positioned 2 cm above the surface of the water. The column was externally marked to indicate three compartments: an upper compartment (U) 12 cm in height, a middle compartment (M) of 10 cm and a lower compartment (L) of three cm in height. Three to four hours before the experiment, the test animals (n =10 females in the second adult instar of a given clone) were placed in dechlorinated tap water in the culture room to acclimatize to this medium. Five minutes before the start of the experiment, the animals were inoculated on the column and placed in the dark box for dark adaptation. After five minutes, the light was turned on. At five minutes and every minute thereafter until the 10^th^ minute, the number of animals in the different compartments was counted. The experiment ended at 10 minutes. The phototactic index was calculated as the number of animals in the upper compartment minus the number of animals in the lower compartment divided by the total number of animals [(U-L)/(U + M + L)] averaged over the five observations.

### QTL mapping

To identify QTL that contribute to variation in life history traits, we performed Haley–Knott regressions and interval-mapping analyses as implemented in R/qtl [49, 51]. Parametric analyses were conducted using a step size of 0.5 cM, but were cross-validated against a nonparametric approach if deviations from normality were suspected. For each F2 clone, the average trait values were used in the linkage mapping. To establish the genome-wide significance level for association between markers and phenotypes, we used 10,000 permutation tests with significant (α =0.05) and suggestive (α =0.1) QTL identified at LOD scores 3.78 and 3.44 respectively. Finally, the effect size (assuming both additive and dominance) and percentage of phenotypic variance explained by each QTL was estimated using the fitqtl() function in R/qtl.

The proportion of phenotypic variation that was explained by *Daphnia* genotype – equivalent to broad-sense heritabilities – was estimated within a Bayesian MCMC framework using the R package MCMCglmm (Hadfield 2010). Non-informative parameter expanded priors were used for the random effect (*Daphnia* genotype), and models were run for 200,000 iterations, with a burn in period of 25,000 and a sampling interval of 50.

## Results

### Genome size estimation with flow cytometry

The estimates for genome size for *D. pulex arenata* is 0.193 pg (range 0.1878 – 0.2087, n = 7), corresponding to 189 mb (range 184 to 204), which is close to the estimated 200 mb resulting from the genome draft for this species [22]. The estimates for *D. magna* clones Xinb3 and Iinb1 are 0.246 (0.228 – 0.262, n = 10) and 0.240 (0.229 – 0.246, n = 6), respectively. This corresponds to 241 mb (223–256) and 235 mb (224–242), respectively, which is not significantly different (t-test, p = 0.25), but significantly larger than the genome size of *D. pulex arenata* (t-test, p < 0.001). In the following we use the overall mean of 238 mb genome size for *D. magna*.

### Genetic map

**Figure 1:**
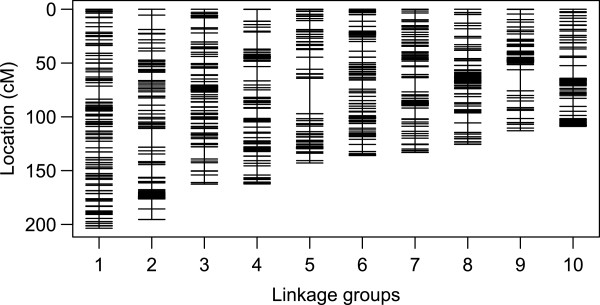
**Linkage groups denoted by vertical lines and arranged in order of decreasing mapping length.** Horizontal lines indicate individual markers. In case multiple markers map to the same location, only one line is shown.

**Figure 2:**
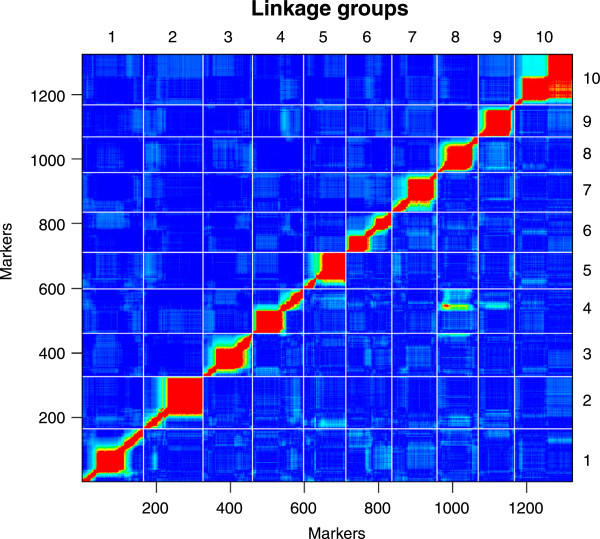
**Heat map of pair wise recombination fractions (above diagonal) and LOD scores (below diagonal) for all marker combinations.** Red indicates high values, and blue indicates low values. Each linkage group has one region or, in the case of group 10, two regions that show strong clustering of SNP markers, indicated by low recombination values and high lod scores. This likely reflects the positions of centromeres, which are known to reduce recombination in their surrounding areas.

**Figure 3:**
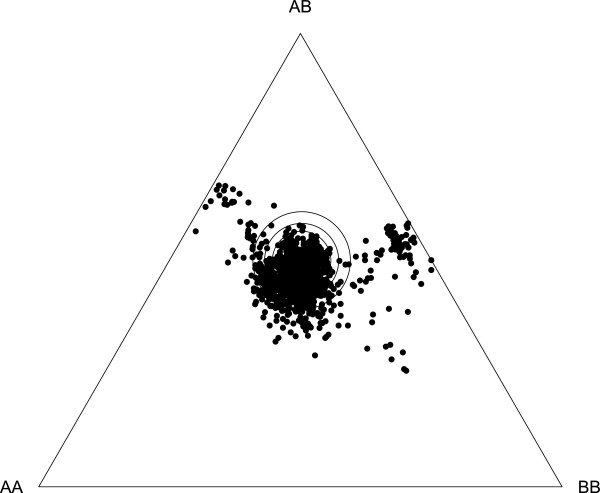
**De Finetti diagram for genotype frequencies of all SNPs used for the map.** The three large central rings show the significance limits for Chi square values of 0.05, 0.01 and 0.001 for deviation from Hardy Weinberg expectations. Ideally (i.e. without an transmission ratio distortion), all markers would fall inside the rings. SNPs falling below the centre show a heterozygote deficiency, while SNPs falling close to the left and the right margin of the triangle show a homozygote deficiency. Markers around the two previously mapped infertility loci show strong homozygote deficiency for one of the two parental alleles each, while the majority of the other markers show a slight heterozygote deficiency. Genotypes are German **(AA)**, Finnish **(BB)** and heterozygotes **(AB)**.

The SNP array typing of the F2 clones resulted in rather low data quality. As a consequence only 8% of the SNP on the array passed our stringent filter criteria. A total of 409,529 genotypes were successfully typed from 353 F2 clones and 1,324 loci. On average, each of these loci provided usable genotypes for 309 F2 clones. All our markers showed detectable linkage to other markers, yielding ten linkage groups ranging in length from 108.9 cM to 203.6 cM (Tables 1 and 2, Figures 1 and 2, supplementary material in Additional files 1 and 2). The total length of all linkage groups was 1,483.6 cM (Kosambi corrected). The average interval between the markers was 1.13 cM, with 95% of all markers within 5 cM of each other, and 71% closer than 1 cM. Associations were revealed between regions in the fourth and the eight linkage groups due to known infertility alleles [27] (see below), which increased the percentage of heterozygous loci (homozygote deficiency) locally and caused high LOD scores without the corresponding recombination fractions (Figure 2). These regions showed significant transmission ratio distortions (TRD) over a length of about 100 cM, affecting the transmission of many markers on the two linkage groups (Figure 3, the two clouds of points to the upper left and right of centre; supplementary material in Additional file 1). In addition to the homozygote deficiencies in these two regions, other substantial parts of the genome showed slight heterozygote deficiencies (Figure 3). Overall, TRD were detected at 43% (p < 0.05, Chi-square test for critical limit; 30% at p < 0.01; 21% at p < 0.001) (Figure 3 and Additional file 1: Table S1) of all SNP markers. If we exclude TRD due to the homozygote deficiency at the two infertility loci, this number drops to 35% (21% at p < 0.01; 11% at p < 0.001). The overall pattern of TRD is identical to the first generation linkage map by Routtu et al. [27].

**Table 1:** **Summary statistics of the ten linkage groups for the SNP-based map of** ***D. magna***

Linkage group	Number of markers	Length (cM)	Average spacing (cM)	Maximum spacing (cM)
1	165	203.6	1.2	12.1
2	161	195.4	1.2	17.4
3	134	162.6	1.2	11.3
4	138	162.4	1.2	15.9
5	113	142.9	1.3	32.9
6	124	136.0	1.1	9.0
7	122	133.2	1.1	13.1
8	111	125.6	1.1	9.3
9	99	112.9	1.2	19.6
10	157	108.9	0.7	11.7
Total	1324	1483.6	1.1	32.9

**Table 2:** **Comparison of genome features, map design features and derived estimates for three** ***Daphnia*** **genetic linkage maps**

	***D. magna*** SNP-based map	***D. magna*** VNTR map [27]	***D. pulex*** VNTR map [26]
Number of chromosomes (from cytological work)	10	10	12
Genome size estimate	238 mb	238 mb	190 mb
Number of markers used for map	1324	109	185
Number of F2 clones used for map	353	214	129
Number of linkage groups recovered	10	10 (+ several minor groups)	12
Map length [cM]	1483.6	1692.5	1206
Average length of marker interval	1.13 cM	15.1 cM	7 cM
Linkage group size range	109 – 203.6 cM	31 – 288 cM (without minor groups)	7 – 185 cM
Physical length/cM	160 kb	141 kb	133 kb
Percentage of markers with transmission ratio distortion	43% (8% homozygote deficiency and 35% heterozygote deficiency)	33% (mainly heterozygote deficiency)	21% (mainly homozygote deficiency)

The two *D. magna* maps are based on the same genetic cross.

Although the average interval between adjacent markers was 1.1 cM, part of the markers did not contribute to the resolution of the map due to clustering without recombination events. However, clustered SNP markers without recombination still contain valuable information, as each scaffold and contig with a mapped SNP marker can be placed in a location to assemble the *D. magna* genome. The size distribution of the marker interval was strongly skewed with many small intervals and only a few large intervals. Intervals >9 cM were found in all linkage groups, with the five largest intervals being 33 cM (LG 5), 19.6 cM (LG 9), 17.5 cM (LG 2), 16 cM (LG 4) and 13.4 cM (LG2). Many factors could account for these regions, e.g. chance events, artefacts of the mapping procedure, long regions of low sequence complexity in the genome, the selection procedure of the SNPs, and recombination hot spots. Large intervals have also been found in the *D. pulex* genetic map [26] and other crustacean maps [4, 6, 52]. On the other hand, each *D. magna* linkage group had one or two regions with very strong linkage disequilibrium (Figures 1 and 2), as indicated by tightly clustered markers. This clustering might be caused by loci that are closely linked physically, but may also indicate regions of reduced recombination around the centromeres. Centromeres, but also possible inversions, reduce recombination rates locally, making it difficult to infer the exact positions of markers within these regions [26].

Because the draft *D. magna* genome (version 2.4) still has many thousands of contigs and scaffolds, it is not possible to compare physical and linkage distance estimates directly, although we can make a rough comparison from the size estimate of the genome. Dividing our genome size estimate (238 mb) by the map length yields an average estimated length of 160 kb/cM, which is slightly longer than the previous estimate of 141 kb/cM for *D. magna* and the 133 kb/cM for *D. pulex*.

The VNTR markers of the first *D. magna* map were not used to construct the second generation map. Mapping the EST based markers from the first generation map [27] on the new map showed that the sequence of the VNTR markers on both maps where largely in agreement with each other (supplementary material in Additional file 3).

### QTL mapping

#### Infertility alleles

Two previously mapped infertility alleles—unviable eggs (UE) and red dwarf (RD) [27]–caused strong homozygote deficiencies among the F2 clones. The distribution of these deficiencies allowed a more precise mapping of these loci than was previously possible. UE caused reductions of BB homozygotes, which originate from the Finnish mother clone. This deficiency is located on linkage group 4 around position 88.385 cM, with the lowest frequency of BB homozygotes (1%) near a marker SNP at position scaffold02065_3570. RD caused a lack of AA homozygotes, which originate from the German father clone. This deficiency is located on linkage group 8, around position 56.988 cM near a marker SNP at contig29432_375, where not a single AA homozygote was found.

#### Life history traits

**Figure 4:**
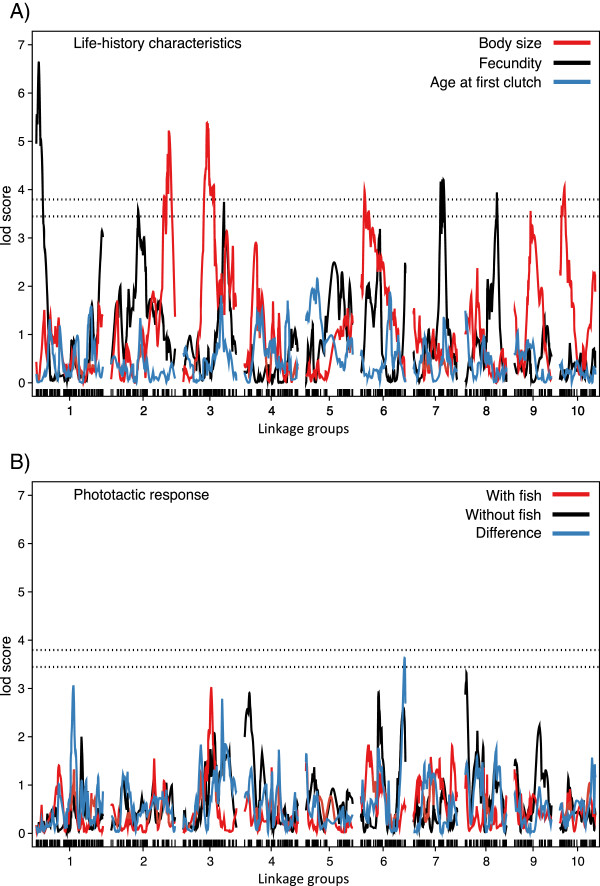
**LOD scores along the 10 linkage groups for variation in A) life history traits and B) phototactic response.** Life history traits include body size (red), lifetime offspring production (black), and age at first clutch (blue). Phototactic response characteristics include the phototactic index in the presence of fish (red), in the absence of fish (black) and the difference between the two for each genotype (blue). Dashed horizontal lines indicate genome-wide thresholds used to identify significant (LOD 3.78) and suggestive (LOD 3.44) QTLs.

**Figure 5:**
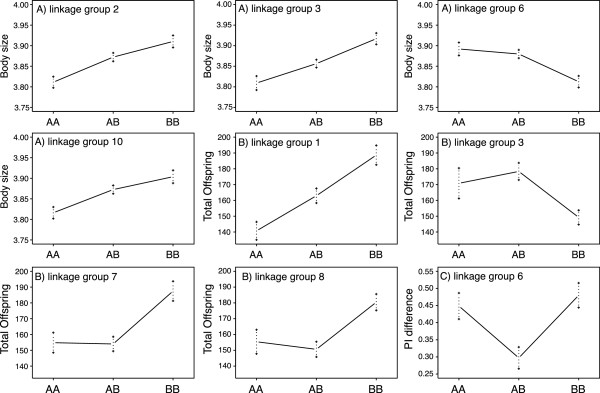
**Phenotypic effect of the identified QTLs on A) body size (linkage groups 2, 3, 6, and 10), B) lifetime offspring production (linkage groups 1, 3, 7 and 8) and C) the difference in phototactic response (linkage group 6).** All error bars represent mean and standard errors. Genotypes are German **(AA)**, Finnish **(BB)** or heterozygotes **(AB)**.

To explore the genetic basis of population divergence, in particular with reference to differences in local predation regimes, we characterize body-size, fecundity and age at first clutch for each of the F2 clones. Body size is largely explained by four loci, mainly additive, each one responsible for between 9 and 12% of phenotypic variance (Figures 4, 5 and Table 3). The effect plots for the markers with the highest LOD scores show that the Finnish alleles are associated with larger body size for three of the four QTLs (on linkage groups 2, 3, and 10) (Figure 5). The QTL on LG 6 shows the opposite pattern, but its contribution to the overall size difference appears small (Figure 5). The overall proportion of genetic variation explained by the *Daphnia* F2-genotypes was 61% for body size, of which only about 42% are explained by the 4 QTLs (Tables 3 and 4).

**Table 3:** **Map positions and descriptive statistics for each of the putative QTLs underlying variation in life history traits and phototactic response for** ***D. magna***

LG	QTL [cM]	95% CI	Length of CI	Marker	LOD	Additive (SE)	Dominance (SE)	% variance
**Body size**								
**2**	176.5	161.6 – 185.6	24.0	scaffold01446_1050	5.2	0.050 (0.010)	0.011 (0.015)	11.7
**3**	72.0	61.5 – 96.6	35.1	contig23286_629	5.4	0.054 (0.011)	-0.006 (0.015)	12.0
**6**	11.1	5.1 – 74.3	69.2	contig28688_84	4.0	-0.040 (0.011)	0.027 (0.015)	9.1
**10**	14.6	3.1 – 24.4	21.3	contig53311_380	4.1	0.045 (0.011)	0.012 (0.015)	9.2
**Lifetime offspring production (= fecundity)**								
**1**	7.32	0.8 – 12.6	11.8	scaffold00772_255	6.6	23.968 (4.199)	-1.767 (6.244)	14.6
**3**	124.7	118.9 – 139.2	20.3	scaffold01201_3880	3.8	-10.091 (5.075)	18.740 (7.363)	8.6
**7**	91.8	76.8 – 101.9	25.1	scaffold01869_1276	4.2	16.472 (4.466)	-16.117 (6.400)	9.5
**8**	96.4	86.9 – 105.7	18.8	scaffold01126_2577	3.9	12.526 (4.623)	-17.220 (6.706)	9.0
**Difference in phototactic response**								
**6**	164.0	126.0 – 136.0	10.1	scaffold00892_2305	3.6	0.016 (0.026)	-0.169 (0.042)	8.2

For each QTL, the location (linkage group (LG) and cM location), 95% confidence interval (CI) of the QTL region, name of nearest marker, lod-score (LOD), effect size (additive and dominance) with standard error (SE) and percent phenotypic variance explained is shown.

**Table 4:** **Statistics describing the phenotypic and genetic variability for the estimates of** ***Daphnia*** **life history and phototactic response**

Trait	Mean	SD	***H*** ^***2***^	95% CI
**Life history traits**				
Body size (mm)	3.856	0.138	0.607	(0.542, 0.657)
Age at first clutch (days)	13.685	2.304	0.191	(0.130, 0.256)
Fecundity	159.367	56.297	0.505	(0.430, 0.569)
**Phototactic response**				
Presence of fish	-0.503	0.386	0.439	(0.363, 0.500)
Absence of fish	-0.104	0.331	0.416	(0.343, 0.481)

Reported are the phenotypic mean and standard deviation (SD), and the posterior mode and 95% credible intervals for the proportion of variation (*H* ^*2*^) that was explained by *Daphnia* F2-genotype. *H* ^*2*^ is equivalent to broad-sense heritability.

The four main candidate loci for *Daphnia* fecundity (= total offspring production) include one additive locus and strong dominance in three loci, with each locus accounting for 9 to 15% of phenotypic variance (Figure 4, 5 and Table 3). The effect plots again show that for three of the four peaks the Finnish alleles are associated with larger fecundity (Figure 5). The peak at LG 3 shows the opposite pattern. The four QTLs together explain 42% of the total variance, while the overall proportion of genetic variation explained by the *Daphnia* F2-genotypes was 50% for fecundity (Tables 3 and 4). Not a single peak was revealed for age at first clutch deposited in the brood pouch (Figure 4). This trait had also the lowest proportion of variation explained by the F2-genotypes (19%, Table 4).

#### Behavioural traits

We tested for QTL corresponding to vertical position in the water in the presence and absence of fish kairomone and also for the difference in vertical position between these two treatments. The former (vertical position in the water) resulted in no significant or suggestive peaks, although about 42% of the total variation was explained by the F2-genotypes (Table 4). The difference between the clonal averages of the positions in the absence and presence of fish (a measure of phenotypic plasticity in predator avoidance behaviour), however, resulted in a suggestive peak on LG 6 (Figure 4, Table 3). According to the effect plot, this signal is produced by a smaller change in position by the heterozygote genotypes at this SNP marker, while the two homozygotes did not differ.

#### Co-localization of QTLs

QTLs for fecundity, body size and the behavioural trait did not co-localize with each other. Nor did they co-localize with the location of the two infertility loci on LG 4 and 8.

## Discussion

The second generation linkage map of *D. magna* presented here greatly improves the previous linkage map of this ecological model species and offers help for the ongoing assembly of the *D. magna* genome project. Furthermore, it provides, as shown here, a tool for mapping phenotypic traits. Flow cytometry revealed that the genome of the two *D. magna* clones used here is with about 238 mb substantially larger than the genome of *D. pulex arenata* (189 mb).

### Genetic map

Two other genetic maps for *Daphnia* have been published: one based on the same mapping panel for *D. magna*[27], the other for *D. pulex*[26]. Table 2 summarizes the main differences in the design of these maps and in the parameters derived from them, showing that the map constructed in this current study is by far the largest, both in terms of markers and F2 clones used, and that it yields better estimates of map parameters. The first mapping project of the North American *D. pulex* was able to construct all 12 chromosomes expected from cytology (2n = 24) [26, 53]. In contrast, the first *D. magna* map resulted in 10 major and several minor linkage groups. Ten groups were expected based on cytological studies of this species [53]. Furthermore, both the first generation *D. magna* and *D. pulex* maps had high variation in linkage group size (>9-fold length difference). The new *D. magna* map yielded not only the predicted ten linkage groups, but also confirmed the cytological observation of relatively little variation in the length of the linkage groups/chromosomes (Table 2; length range: 109 – 203.6 cM). The physical distance estimates of the three Daphnia maps are in good agreement with each other (Table 2).

As was observed in the first generation genetic map of *D. magna*, we found transmission ratio distortion (TRD) in several areas of the linkage map, which caused deviations of expected HW equilibrium in the F2 panel (Figure 3). Two different mechanisms were responsible for this TRD. First, two infertility alleles in the F1 clone, originating from each parental genotype, caused the loss of one homozygote each. TRD around these two loci could be used to map their position to two different linkage groups in the F2 panel. Together, they explain about one fifth of all TRD. Neither of them co-localized with any other QTLs found for the traits mapped in this study, although one infertility allele was closely linked with a QTL for resting egg (ephippia) production in an earlier study [29]. Second, TRD was also apparent for other SNPs; however, in these cases it was due to deficiencies of heterozygotes, visible as a clustering of SNP markers below the centre in Figure 3. Heterozygote deficiency indicates some degree of F2 hybrid breakdown. As the parent clones for our F2 panel are from ecologically distinct populations about 1,500 km apart (ephemeral rock pool habit in Finland vs. carp breeding pond in southern Germany), it is likely that synergistic epistasis acts on genes in different parts of the genome, influencing the parental phenotypes. Co-adapted gene complexes in our F2 panel might have been broken down by recombination, leading to a depression in F2 fitness and an underrepresentation of heterozygotes among the viable F2 clones. While we lost very few F2 clones once the F2 hatchlings from the sexual eggs started reproduction, we cannot exclude selection during the preceding stages, i.e. survival of embryos in resting eggs, embryonic and juvenile development and maturation, all of which might contribute to outbreeding depression [54]. The heterozygote deficiencies seen at several linkage groups were much less drastic than the homozygote deficiencies caused by the two infertility alleles (Figure 3).

Each linkage group showed dense clusters of markers, indicating strong LD (Figure 1). These regions might indicate groups of markers that are tightly linked physically, but may also indicate regions of reduced recombination, as is typical for centromeres. This latter explanation was given for a similar situation in the *D. pulex* linkage map, where a large number of loci were also found clustered at identical positions [26]. The strong LD close to the putative centromeres makes mapping of phenotypic traits difficult due to lack of recombination in these areas. However, none of the QTLs for traits mapped in this or earlier studies using the same mapping panel [29] fall into these regions.

### Mapping of life history and behavioural traits

Our two parent clones for this mapping panel were collected from a fishless rock pool habitat and a carp breeding pond, respectively. Previous studies have reported that *D. magna* adapts locally to the presence of fish through smaller body size, earlier maturation, and a deeper average position in the water [38, 55]. Furthermore, animals from populations with a history of sympatry with fish, are found deeper in the water when exposed to fish kairomone [50]. Quantitative genetic studies have revealed significant broad sense heritabilities for these traits [19, 56–58]. Our phenotypic mapping provides some insight into the genetics of these traits. A pattern consistent with local adaptation is found for body size, where alleles from the fishless Finnish rock pool population were mostly associated with larger body size (Figure 5). QTLs for higher fecundity (Figure 5) are in agreement with the general finding of higher reproductive effort in populations with fish, but higher fecundity is not consistently associated with *Daphnia* from ponds with fish [38, 55]. We found no strong QTLs for the other traits—age at first clutch and position of *Daphnia* in the water column. A potential (0.05 < *P* <0.10) QTL was found for changing position in the water when we compared experimental conditions with and without fish kairomone, but the observed phenotypic pattern did not correspond to our expectation of a stronger change associated with alleles from the population from the fish habitat; instead, heterozygote *Daphnia* tended to show the least response.

Our failure to find QTLs for these traits is not evidence for the absence of genetic variation, as we found for all traits significant genetic variance components among the F2 clones (Table 4). QTL studies are most powerful for phenotypes with strong effects caused by one or few loci, with little sensitivity to environmental variation [59], which is rarely the case. The absence of significant QTLs allows some speculations about the underlying genetic architecture. However, it has to be kept in mind, that with only one clone from each population and data based on a panel from an interpopulation cross, these speculations are rather preliminary. Nevertheless, it is clear, that the traits studied here clearly differ in their underlying genetic architecture. For example, the 8.2% variation explained by the significant QTL detected here for phototactic behaviour (Table 4) suggests that the genetic effect responsible for this difference stems from multiple genes with small effects. De Meester [19] obtained similar estimates in a clonal repeatability analysis and in an offspring-on-midparent regression upon sexual recombination, suggesting that the genetic variation underlying this trait is largely additive in nature. The emerging picture thus is that this trait may represent a case of additive, polygenic inheritance. In contrast, the two phenotypically distinguishable infertility loci, characterized in a previous study [27], could be mapped based on the strong transmission ratio distortion caused by the absence of one homozygote class. Their underlying genetics is simple: each has one locus with two alleles, with no known environmental contributions and no epistatic interactions. For both loci, we also know that they are polymorphic in the natural populations, as the parent clones are heterozygote [27]. For body size and fecundity, we found a more complex pattern with additive and dominant QTLs explaining about 40% of total phenotypic variance, and both traits influenced by multiple QTLs scattered throughout the genome. Thus, our QTL study revealed traits with strongly differing genetic architecture, ranging from single locus effects with dominance but no inter-locus interactions, to typical quantitative genetic traits with many loci and potential interactions. Follow-up studies need to reveal if these genetic architectures also reflect the situation within populations.

The genetic architecture of the traits mapped here appear to be largely independent, and we found no pleiotropic effects, or close physical linkage between loci influencing different traits. Our results thus suggest that any genetic correlation between traits across populations, e.g. body size and fecundity, is maintained by the build-up of inter-population linkage disequilibrium between loci, caused by selection.

Because the SNPs used in our genetic map can be located on the *D. magna* genome draft, it may raise the possibility of fine mapping the QTLs on the genome to identify the exact genes responsible for the phenotypic effect. However, the prospects of fine mapping have to be improved. The current *D. magna* genome draft (version 2.4) still consists of several thousand scaffolds and contigs. The genomic regions of the QTL identified here are, however, more than 10 cM (mostly more than 20 cM), equal to about 1.6 mb. This is far larger than the scaffolds and contigs containing the SNP markers that describe the location of the QTL. Furthermore, the effect sizes of most of the individual QTLs (Figure 5) are not strong enough for fine mapping. Only the QTL for fecundity on linkage group 1 shows a strong effect, but the best marker is located on a scaffold of only 3.68 kb (Table 3) (the confidence interval in this case is 11.8 cM, about 1.9 mb). Thus, fine mapping is not possible until we have a better genome draft, which would allow us not only to locate more SNPs in the mapped region, but would also enable a candidate gene approach. Increasing the density of our genetic map would also help, although this seems not to be a limiting factor at the moment, as we already have about seven times more genetic markers than recombinant lines. Using more recombinant lines would be more likely to substantially improve the mapping statistics. Since we used only 193 F2 clones for phenotyping, but built the map with 353 F2 clones, a first step would be to phenotype the remaining 160 F2 clones. Alternatively, one could repeat the entire study with a different mapping panel, which would allow us to find the QTLs that explain variation in more than one pair of populations and help us fine-map phenotypes by overlapping the QTLs from different studies, as has been done for similar cases [60].

## Conclusions

The new genetic map of *D. magna* presented here only partially fulfilled our aim to produce a high density SNP backbone for the assembly of the *D. magna* genome. With only 8% of the SNPs resulting in data of sufficient quality for genotype calling, the usefulness for the genome assembly is lower than expected. Nevertheless, the number of SNPs is high enough to significantly help in the assembly of the genome. On the other hand, the map proved a powerful tool to analyse the genetic architecture of several traits segregating in the populations of origin. The spectrum of genetic architectures ranges from single locus traits (deleterious recessive mutants for infertility) to complex traits with variable numbers of QTLs and possible interactions among loci. For traits with strong QTL it may be possible in the future to fine map the genes in question.

## Authors’ information

W. Kelley Thomas, Michael E. Pfrender and Dieter Ebert shared senior authors.

## Electronic supplementary material

Additional file 1: **Raw data for the genetic map, sorted according to the results from the linkage mapping.** Scaffolds refer to *D. magna* genome assembly 2.4. Information listed includes the names of the SNP marker; the number of the linkage group; the position of the marker within the linkage group (in cM); the number of AA homozygotes (from the German father clone), AB heterozygotes, and BB homozygotes (from the Finnish mother clone); total number of genotypes called for this marker; expected number of AA, AB and BB genotypes (assuming 0.25:0.50:0.25 segregation ratios); and the p-value of Chi-square tests for a difference between observed and expected genotype frequencies (p < 0.05 indicates significant transmission ration distortion). (XLSX 192 KB)

Additional file 2: **FASTA file with the SNP sequence tags used for the genetic map.** Scaffold labels refer to *D. magna* genome assembly 2.4. (ZIP 25 KB)

Additional file 3: **Compilation of the EST-based markers from Routtu et al.** [27] **combined with the new SNP map marker map.** Information listed includes the names of the SNP marker and the EST derived marker (short labels starting with a P), respectively; the number of the linkage group, and the position of the marker within the linkage group (in cM). (XLSX 85 KB)
